# Spirituality and Sexuality: Not Necessarily a Binary Choice for LGBTQ+ People

**DOI:** 10.1093/geroni/igaa057.2359

**Published:** 2020-12-16

**Authors:** Michael Thomas

**Affiliations:** Brunel University London, Uxbridge, England, United Kingdom

## Abstract

This paper reports on a qualitative study on the impact of marriage and civil partnerships for lesbian, gay and bisexual (LGB) couples. Drawing on data from 50 dyad interviews in the UK, US and Canada, the paper investigates the ways in which couples make sense of spirituality in the context of a stigmatised sexuality. For some, the task of arranging a wedding or civil partnership ceremony provided a powerful reminder of their exclusion from mainstream religious denominations. This sense of stigma (Goffman, 1963) was also present in later life, when the lack of social esteem granted to same-sex relationships gave rise to a sense of disenfranchised grief (Doka, 1989). Whereas some participants tended to frame sexuality and spirituality as a kind of binary choice, others resisted this marginalisation from religious and spiritual activities, even if this meant finding a personal sense of spirituality beyond the confines of organised religion.

